# Support from a Best Friend Makes People Eat Less under Stress: Evidence from Two Experiments

**DOI:** 10.3390/nu15183898

**Published:** 2023-09-07

**Authors:** Mingyue Xiao, Yijun Luo, Weiyu Zeng, Hong Chen

**Affiliations:** 1 Key Laboratory of Cognition and Personality, Ministry of Education, Faculty of Psychology, Southwest University, Tiansheng Road No. 2, Beibei District, Chongqing 400715, China; 2 Research Center of Psychology and Social Development, Faculty of Psychology, Southwest University, Tiansheng Road No. 2, Beibei District, Chongqing 400715, China

**Keywords:** social support, acute stress, eating behavior, negative affect, self-efficacy, reward sensitivity

## Abstract

When experiencing acute stress, individuals often turn to eating for comfort, as it provides a sense of satiety and satisfaction that can temporarily alleviate the stressful condition. However, this may increase the risk of obesity, diabetes, cardiovascular disease. In this study, we conducted two behavioral experiments to investigate the effectiveness of social support in reducing stress-induced overeating and the mediative role of negative affect and self-efficacy (Experiment 1), as well as the role of reward sensitivity (Experiment 2). Acute stress was induced using a speech preparation task and then participants were asked to regulate their emotions and cognition, either alone or with the help of pictures and supportive sentences provided by a best friend or stranger. Participants in Experiment 1 then completed the food choice task, and participants in Experiment 2 completed the food incentive delay task and the bogus tasting task. The results of both experiments consistently showed that participants who received support from their friends reported lower levels of perceived stress, chose fewer food portions, and consumed fewer snacks during acute stress, compared to the other three groups. Further mediation analysis using the process macro revealed that the differential influence of social support on the choice of high-calorie foods was due to decreased negative affect and increased self-efficacy. This study provides valuable insights for the development of therapeutic interventions for clinical eating disorders.

## 1. Introduction

Many people cope with stress by increasing their food intake [1], particularly by consuming foods that are high in calories, fat, or sugar [2,3,4]. Given that excessive emotional eating has been strongly associated with obesity [5] and eating disorders [6,7], it is crucial to explore preventive factors of this unhealthy eating behavior and the underlying mechanisms involved.

Social support is defined as the resources provided by one’s social network with the intention of enhancing coping ability [8]. Cross-sectional and longitudinal studies have consistently shown that social support is a crucial factor in preventing the development and maintenance of stress-induced overeating, and in promoting healthy eating behaviors [9,10]. We propose that this influence occurs mainly through the effect of social support on negative affect, self-efficacy, and reward sensitivity under stress.

Describing different forms of social support, Cobb distinguished between “emotional support” and “cognitive support” [11]. Emotional support, which conveys love, acceptance, and a sense of belonging, could foster positive changes in one’s self-identity [11]. The interpersonal model of binge eating [12,13] proposed an indirect relationship between interpersonal problems and eating disorder symptoms via negative emotions. Cross-sectional and longitudinal studies have consistently demonstrated that negative emotions mediate the relationship between social support and disordered eating [14,15]. On the other hand, cognitive support, which enhances an individual’s sense of competence and efficacy, can encourage them to cope with a given problem [11]. Research on the role of cognitive support in eating disorders has emphasized the importance of self-efficacy [16]. Previous research has demonstrated a close relationship between social support and self-efficacy [17]. In fact, family and friends could help to improve an individual’s self-efficacy by demonstrating effective coping strategies for dealing with adversity [17], thereby reducing perceived stress and the likelihood of engaging in maladaptive eating behaviors.

Furthermore, acute psychological stress may lead to regional changes in the functioning of reward processing circuits, resulting in behaviors such as excessive reward seeking [18]. From an evolutionary perspective, both food [19] and social connection [20] are essential for human survival and should be considered subsistence resources [21]. According to the fundamental principle of the self-regulation of motivation and action [22], cues that signal the abundance (or deprivation) of one of the subsistence resources decrease (or increase) the incentive value of others [23]. For example, participants primed with social distancing consumed more ice cream in a taste test [21], and those led to believe that they were likely to be alone later in life donated less money than those who anticipated a future of belonging or those in a control group [24]. We believe that this substitution effect may also reduce stress-induced overeating through the effect of social support on reward sensitivity.

The aim of this study was to investigate the role and mechanism of social support in reducing stress-induced overeating through two behavioral experimental studies. Specifically, in four groups, participants received a best friend’s support, stranger’s support, regulated alone, and went without regulation [25]. We used a combination of emotional and cognitive support to manipulate social support, in order to understand its effectiveness [26]. In addition, we provided participants with high- and low-calorie food choices to investigate the effect of stress conditions on food type. We hypothesized that (1) social support from a best friend would significantly contribute to the reduction of perceived stress, accompanied by a decrease in negative affect, an increase in self-efficacy, and ultimately a reduction in food choice; (2) social support can function as a substitute for food rewards in satisfying individuals’ reward requirements when facing stress, leading to a reduction of food reward sensitivity and stress-induced actual food consumption.

## 2. Methods

**Figure 1:**
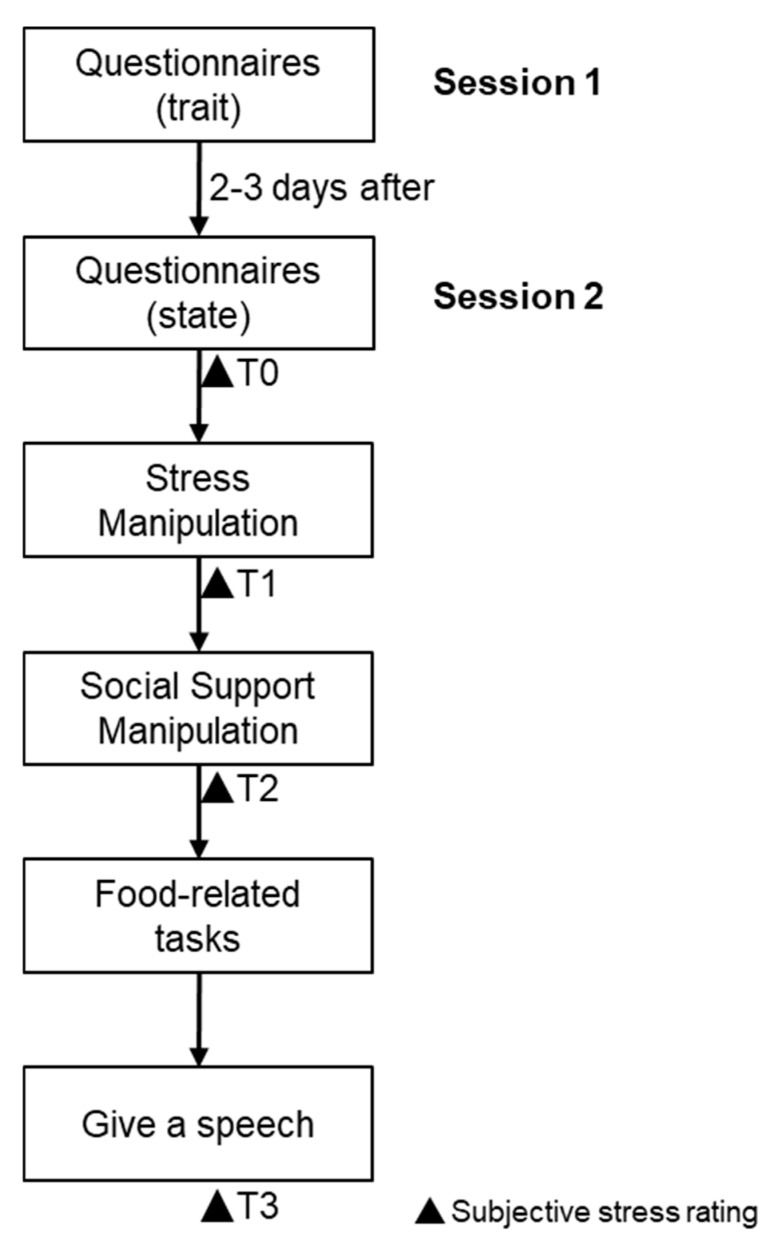
The present study flow. Note: T0, Baseline; T1, post-stress induction; T2, post-support manipulation; T3, post-speech presentation.

Here we report two studies: an experiment to investigate the mechanisms of social support on food choice through negative affect and self-efficacy (Experiment 1), and an experiment to investigate the mechanisms of social support on actual food consumption through reward sensitivity (Experiment 2). The two experiments used similar experimental paradigms and are therefore described together in this section. Session 1 was identical for both experiments, while Session 2 comprised different food-related behavioral tasks (Figure 1). Ethical approval for this study was granted by the Ethics Committee of the University (No. H22033), and all procedures involved were in accordance with the sixth revision of the Declaration of Helsinki.

### 2.1. Participants

***Experiment 1:*** Participants (N = 140) were recruited via advertisements distributed across campus and a digital research participation platform. Due to technical problems with data recording, only N = 138 (113 females; mean age = 20.87 years, SD = 1.58) were accepted in the analyses, including 34 participants in the Friend group, 37 participants in the Stranger group, 33 participants in the Decrease group, and 34 participants in the Look group.

***Experiment 2:*** Participants were recruited via advertisements distributed across campus and a digital research participation platform. To ensure a balanced sample distribution, we excluded participants whose initial stress levels were either too high or too low, in order to match the four experimental groups. The participants N = 136 (110 females; mean = 20.92 years, SD = 1.49) were recruited, including 36 participants in the *Friend* group, 35 participants in the *Stranger* group, 32 participants in the *Decrease* group, and 33 participants in the *Look* group. It should be noted that in order to optimize resource utilization, participants who were part of the *Stranger* and *Decrease* groups in Experiment 1 were also included in the experimental procedures of Experiment 2. Specifically, they completed the Food Incentive Delay (FID) task prior to making their food selection and performed the food eating task prior to the speech task. In contrast, new participants were recruited for the *Friend* and *Look* groups.

### 2.2. Procedure

In the Session 1, participants were randomly assigned to one of four groups: *Friend*, *Stranger*, *Decrease*, and *Look* group. All participants completed a set of questionnaires (see Method S1 in Supplementary Materials). Participants in the *Friend* group were instructed to bring along their best friend, which was explicitly defined as someone with whom they did not have a sexual relationship. For the other three groups, they were asked to come to the lab alone. The best friends in the friend support group were asked to provide five supportive statements for the participants that could be utilized in emotionally challenging situations. It was explained to the friends that these statements would be presented to the participants in Session 2. But in fact, in Session 2, everyone saw the same sentences (see Method S2 in Supplementary Materials).

To minimize the risk of participants guessing the experiment’s purpose and to mitigate the impact of trait questionnaires on the results, Session 2 was conducted 2–3 days following the completion of Session 1. In addition, participants were informed that Session 2 consisted of several distinct subtasks, each unrelated to the others. Following the experiment, each participant underwent an interview, revealing that no one had correctly guessed the study’s purpose as investigating the impact of social support on stress-induced overeating. Specifically, in Session 2, participants first reported their current stress level, positive and negative emotion level, hunger level, and how long it had been since they last ate (baseline, T0). Participants then completed a stress manipulation task and again reported their stress level (post-stress induction, T1). Each of the four groups completed different social support manipulation tasks and then reported their feelings of stress (post-support manipulation, T2). Immediately afterwards, they completed a food portion selection task (Experiment 1) or an FID task and a bogus tasting task (Experiment 2). Finally, participants were taken to another room, completed a presentation, and took a state of stress measurement at the end of the presentation, which reflected stress levels after the stressor had been removed (post-speech presentation, T3).

### 2.3. Experimental Manipulations

***Stress manipulation.*** We manipulated the perceived stress level via a speech task, which is a commonly used, effective stress induction strategy [15,27]. When they completed the state questions, the participants were asked to spend five minutes preparing a presentation on the topic of “global warming”. Two judges (one male and one female) were present to watch and evaluate their speech presentation online via video conference. The two judges were impersonated by two of the main experimenters, who were asked to keep their faces expressionless during the presentation.

***Social Support Manipulation.*** We manipulated the perceived support level via a social support manipulation task [25]. Participants in the social support (*Friend* and *Stranger*) group were presented with a picture of their best friend (*Friend*) or an experimenter (*Stranger*) on the screen with a supportive statement below the picture, and were asked to cope with the stressful thoughts related to the speech with the support they had received. In the *Decrease* group, participants viewed a scrambled image along with the instruction to actively regulate their emotions and cognition. Importantly, no specific tactic was instructed, just a general statement to regulate their emotions or cognition was presented. Importantly, participants were told not to substitute negative emotions with positive emotions as this would result in distraction from negative emotions rather than a reappraisal of the depicted situation [28]. This group was non-social and required intrapersonal emotion regulation. In the *Look* group, participants were first presented with a scrambled image and underneath it the instruction to view the following aversive image attentively and allow themselves to experience/feel any emotional responses which it might elicit without manipulating their emotions. This established the control condition. The material details of social support manipulation are described in Method S2. Emotional state (1 = very negative, 5 = very positive) and confidence for subsequent speech (1 = very little, 5 = very much) were measured in every trial. State of stress perception and perceived supportiveness were measured after the social support manipulation.

### 2.4. Food-Related Tasks

***Experiment 1: Food portion choice task.*** A food portion choice task was used in this study [29]. For each separate trial, the participants were asked to choose how much food they wanted to eat based on their current state. The instructions were as follows: “Each of the following trials consists of one kind of food with a portion size of 0 to 4. Please choose the appropriate portion size you want to eat based on how you currently feel. Press 0 for none, 1 for one, 2 for two, 3 for three, and 4 for four”. In total, this study comprised 20 high-calorie (e.g., chips) and 20 low-calorie foods (e.g., tomatoes).

***Experiment 2: Food Incentive Delay (FID) task.*** This study employed a modified version of the FID task proposed by Simon [30]. During each trial, the participants were presented a geometric figure (*cue*), then waited for a fixed interval (*anticipation*), and then responded to the direction of the arrow with a button press (*reaction*). Immediately after the response, feedback appeared documenting whether the participant had won a food reward at that point (*feedback*) and chosen the food portion size (*choice*). The participants were told that they would win high-calorie foods (e.g., chips), low-calorie foods (e.g., tomatoes), and neutral rewards (e.g., a clip) based on their reaction performance, which was signaled by the square, triangle and circle cues, respectively. Because each cue matched a class of food/object, the participants had to learn and remember the picture of the food/object corresponding to each cue before the task began. Only those who reached 90% accuracy were considered to have learned the matching rules and could proceed with the formal trials, which included 60 trials, with 20 trials for each cue. Food reward sensitivity was defined as the reaction times to the target in the food reward trials. Shorter reaction times reflected higher reward sensitivity [31,32].

***Experiment 2: Bogus tasting task.*** As a behavioral measure of high/low-calorie actual food consumption, participants were offered a bowl of tomato-flavored potato chips and a bowl of tomatoes. To decrease the influence of social desirability during this eating, the participants were left alone and instructed to eat as much of the tomatoes and chips as they liked in the next ten minutes to accurately appraise these foods. At the end of the experiment, the experimenter returned to administer a manipulation check. Their consumption was later documented, and further analyzed as an actual consumption for either high- or low-calorie foods.

## 3. Results

### 3.1. Experiment 1 Results

***Descriptive statistics.*** The statistical test power of the present samples was calculated using G*Power version 3.1 [33]. A two-factor mixed design with a sample size of 138 and an effect size of 0.3 has a statistical test power of over 90% (1-β power). The description of gender, age, BMI, hunger, emotional eating, trait perceived social support, trait perceived stress, trait self-efficacy, and baseline emotional status are shown in Table 1. Results of the one-way ANOVA analysis indicated that except age (*p* < 0.05), trait/state of negative emotion (*ps* < 0.05), and cognitive restraint (*p* = 0.06), differences between the groups were not significant (*ps* > 0.05). We then included variables with significant inter-group differences as covariates in subsequent analyses.

**Figure 2:**
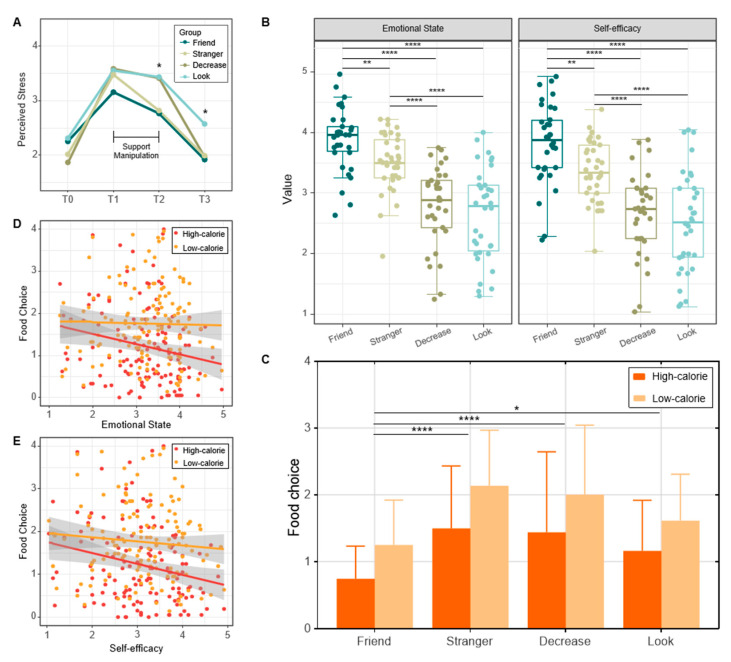
(**A**) Perceived stress in response to the laboratory stressor at four time points. Participants in the *Friend* and *Stranger* groups reported less perceived stress compared to the *Decrease* and *Look* groups at T2, and stress of the *Look* group was higher than other three groups at T3. (**B**) Results of the negative affect and self-efficacy ratings following each trial of the experiment. Participants felt less negative affect (1 = very negative, 5 = very positive) and more self-efficacy (1 = very little, 5 = very much) in the *Friend* and *Stranger* groups compared to the *Decrease* and *Look* groups. The regulation with the help of a friend was most effective. (**C**) Results of the high- and low-calorie food choices. Participants chose less high-calorie and low-calorie foods in the *Friend* group compared to the other three groups. (**D**) Relationship between negative affect and high/low-calorie food choice. Less negative affect was associated with less high-calorie food choice, but not low-calorie food choice. (**E**) Relationship between self-efficacy and high/low-calorie food choice. More self-efficacy was associated with less high-calorie food choice, but not low-calorie food choice. Note: * *p* < 0.05; ** *p* < 0.01; **** *p* < 0.001; T0, baseline; T1, post-stress induction; T2, post-support manipulation; T3, post-speech presentation. Error bars indicate standard error of the mean.

***Manipulation test for acute stress***. To test the effect of stress induction, we used a repeated-measures ANOVA to compare differences between the experimental groups (*Friend*, *Stranger*, *Decrease*, and *Look*) over time (baseline (T0), post-stress induction (T1), post-support manipulation (T2), and post-speech presentation (T3)). Trait perceived stress and trait/state emotions were used as covariates. The results revealed a significant interaction effect, *F*(3, 129) = 3.061, *p* < 0.005, *η*^2^ = 0.066, and showed a significant main effect of measurement time, *F*(3, 129) = 128.546, *p* < 0.001, *η*^2^ = 0.752, and a significant main effect for group, *F*(3, 129) = 7.252, *p* < 0.001, *η*^2^ = 0.144. Post hoc analysis results indicated that the stress level after stress induction (M = 3.44, SD = 0.07) was significantly higher (*p* < 0.001) compared to the baseline level (M = 2.11, SD = 0.04), indicating that stress induction was successful. Simple effects analysis showed that the differences among the four groups were not significant at T0 and T1, but the stress level of the *Friend* and *Stranger* group were significantly lower than those of the *Decrease* and *Look* group at T2, and *Friend*, *Stranger*, and *Decrease* groups were significantly lower than those of the *Look* group at T3 (Figure 2A).

***Manipulation test for social support.*** To assess whether the manipulation of social support was successful, we conducted a one-way ANOVA analysis, with support condition as an independent factor. The results showed that participants in the *Friend* (M = 4.03, SD = 0.52) and *Stranger* (M = 3.73, SD = 0.80) groups felt more support than those in the *Decrease* (M = 2.12, SD = 0.86) and *Look* (M = 2.62, SD = 0.99) groups (*F* = 42.393, *p* < 0.001).

***Food choice.*** Repeated-measures ANOVA revealed no significant interaction effect, *F*(3, 129) = 0.102, *p* = 0.959, *η*^2^ = 0.002; a significant main effect of food type, *F*(1, 129) = 57.834, *p* < 0.001, *η*^2^ = 0.310; and a significant main effect for group, *F*(3, 129) = 9.308, *p* < 0.001, *η*^2^ = 0.178. Specifically, participants chose significantly fewer high-calorie foods (M = 1.21, SD = 0.07) than low-calorie foods (M = 1.75, SD = 0.07) (*p* < 0.001). Participants in the *Friend* (M = 0.93, SD = 0.13) group chose less food than those in the *Stranger* (M = 1.86, SD = 0.12), *Decrease* (M = 1.68, SD = 0.13), and *Look* (M = 1.45, SD = 0.13) groups (*ps* < 0.001) (Figure 2C).

***Negative affect.*** To examine the difference of negative affect among groups, we conducted a one-way ANOVA analysis, with support conditions as an independent factor. The results showed that participants in the *Friend* (M = 3.89, SD = 0.51) and *Stranger* (M = 3.51, SD = 0.47) groups felt less negative affect than those in the *Decrease* (M = 2.79, SD = 0.66) and *Look* (M = 2.65, SD = 0.76) groups (*F* = 31.701, *p* < 0.001) (the lower the score, the higher the level of negative affect). The help of a friend for emotional regulation was more effective than a stranger (*p* < 0.01) (Figure 2B). In addition, there was a significant correlation between emotional state and high-calorie food choice, i.e., the higher the emotional state score (less negative affect), the lower the high-calorie food choice. We did not find this relationship in the low-calorie food choice (Figure 2D).

***Self-efficacy.*** To examine the difference in self-efficacy between groups, we conducted a one-way ANOVA analysis, with support conditions as an independent factor. The results showed that participants in the *Friend* (M = 3.82, SD = 0.65) and *Stranger* (M = 3.36, SD = 0.51) groups felt more self-efficacy than those in the *Decrease* (M = 2.67, SD = 0.68) and *Look* (M = 2.51, SD = 0.83) groups (*F* = 27.862, *p* < 0.001). The help of a friend for cognitive regulation was more effective than a stranger (*p* < 0.01) (Figure 2B). In addition, there was a significant correlation between self-efficacy and high-calorie food choice, i.e., the more self-efficacy for the presentation, the lower the high-calorie food choice. We did not find this relationship in the low-calorie food choice (Figure 2E).

***Mediation analysis.*** We used a mediation model to test the effect of social support on food choice. Specifically, our model included perceived social support as the independent variable, negative affect or self-efficacy as the mediating variable, and the portion size choice of high-calorie foods as the dependent variable. Results revealed a significant mediation of negative affect on the relationship between social support and high-calorie food choice [ab = −0.150, 95%CI: −0.31, −0.03] and a significant mediation of self-efficacy on the relationship between social support and high-calorie food choice [ab = −0.15, 95%CI: −0.29, −0.05].

### 3.2. Experiment 2 Results

***Descriptive statistics.*** The description of gender, age, BMI, hunger, emotional eating, trait perceived social support, trait perceived stress, trait self-efficacy, and baseline emotional status are shown in Table 2. Results of the one-way ANOVA analysis indicated that except for age, uncontrolled eating, and cognitive restraint (*ps* < 0.005), the differences between the groups were not significant (*ps* > 0.05). We then included variables with significant inter-group differences as covariates in subsequent analyses.

***Manipulation test for acute stress***. To test the effect of stress induction, we used the same analysis as in Experiment 1. The results revealed a significant interaction effect, *F*(3, 127) = 5.909, *p* < 0.001, *η*^2^ = 0.122, and showed a significant main effect for measurement time, *F*(3, 125) = 95.371, *p* < 0.001, *η*^2^ = 0.696, but no significant main effect for group, *F*(3, 127) = 1.266, *p* < 0.289, *η*^2^ = 0.029. Post hoc analysis results indicated that the stress level after stress induction (M = 3.27, SD = 0.07) was significantly higher (*p* < 0.001) compared to the baseline level (M = 1.99, SD = 0.05), indicating that stress induction was successful. Simple effects analysis showed that the differences among the four groups were not significant at T0, T1, and T3, but the stress level of the *Friend* group were significantly lower than those of the *Stranger*, *Decrease,* and *Look* groups at T2.

***Manipulation test for social support.*** To assess whether the manipulation of social support was successful, we conducted a one-way ANOVA analysis, with support condition as an independent factor. The results showed that participants in the *Friend* (M = 3.94, SD = 0.71) and *Stranger* (M = 3.80, SD = 0.68) groups felt more support than those in the *Decrease* (M = 2.25, SD = 0.88) and *Look* (M = 2.61, SD = 0.78) groups (*F* = 38.726, *p* < 0.001).

**Figure 3:**
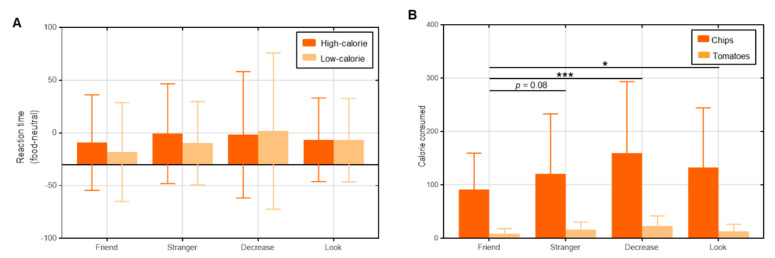
(**A**) Results of the reward sensitivity. There was no significant difference in reaction time (food minus neutral) among the groups and between the food types. (**B**) Results of the actual consumption. Participants in *Friend* group ate less calories than those in *Stranger, Decrease* and *Look* groups. Note: * *p* < 0.05; *** *p* < 0.005. Error bars indicate standard error of the mean.

***Food reward sensitivity.*** To exclude the effect of the general reward sensitivity of individuals, we measured reward sensitivity to food as follows: food cue reaction time minus neutral objects reaction time [30]. A repeated-measures ANOVA revealed no significant interaction effect, *F*(3, 128) = 0.489, *p* = 0.690, *η*^2^ = 0.011; no significant main effect of food calories, *F*(1, 128) = 0.917, *p* = 0.340, *η*^2^ = 0.007; and no significant main effect for group, *F*(3, 128) = 0.839, *p* = 0.475, *η*^2^ = 0.019 (Figure 3A).

***Actual consumption.*** We then multiplied the actual amount of high/low-calorie food eaten (g) by their unit calories (potato chips: 5.44 kcal/g; tomatoes: 0.25 kcal/g), respectively, to obtain the actual calories of high/low-calorie food eaten. Results revealed no significant interaction effect, *F*(3, 125) = 2.022, *p* = 0.114, *η*^2^ = 0.046; a significant main effect of food type, *F*(1, 125) = 115.944, *p* < 0.001, *η*^2^ = 0.555; and a significant main effect for group, *F*(3, 125) = 3.621, *p* < 0.05, *η*^2^ = 0.080. Specifically, participants consumed more calories from high-calorie food (M = 125.95, SD = 9.08) than from low-calorie food (M = 15.16, SD = 1.19) (*p* < 0.001). Participants in the *Friend* (M = 45.92, SD = 9.35) group ate fewer calories than those in the *Decrease* (M = 91.03, SD = 10.38, *p* < 0.005) and *Look* (M = 74.47, SD = 10.72, *p* < 0.05) groups with statistical significance, while those in the *Stranger* (M = 70.80, SD = 9.95, *p* = 0.08) group showed marginal significance (Figure 3B).

## 4. Discussion

Based on the notion that stress induces eating behaviors and that social support might buffer that effect, we conducted two laboratory experiments. The results of both studies consistently indicated that participants with a friend’s support reported a lower level of perceived stress and a reduction in food choice or consumption under acute stress, compared to those with stranger’s support, regulating alone, or without regulation. This finding is consistent with previous studies that found higher perceived social support was associated with lower perceived stress [15] and lower cortisol responses [2]. Importantly, Experiment 1 provided mediation evidence for emotional state and self-efficacy as our underlying processes: increased emotional state (i.e., decreased negative affect) and self-efficacy negatively predicted the choice of high-calorie foods. Experiment 2 revealed that social support from friends resulted in a reduction of calories consumed, which extends the effect of social support beyond food portion selection to problematic eating behaviors during actual food consumption.

More importantly, in Experiment 1, friend support was more effective than the other three conditions in increasing emotional state and self-efficacy, and reducing food choice. These findings suggest that while both friend and stranger (e.g., experimenters) support reduced participants’ perceived stress under acute stress, the support from friends played a unique role. This may be because friends were socially closer than strangers and participants felt more loved and respected, leading to the decrease of negative emotions, and improved the motivation of action. Previous research has found that support from strangers can elicit negative emotions and activate the amygdala, which does not contribute to reducing perceived stress under acute stress [25]. Similarly, some researchers have noted that the effects of social support from health professionals may be limited and short-lived compared to support from patients’ natural support networks [34]. This may be due in large part to the non-reciprocal relationship between patients and health professionals. The present study speculates accordingly that the involvement of real friends (or even family members) may be more effective in interventions with clinical samples than those involving only staff members. Close social relationships, in other words, may have a more direct impact on changing eating behaviors through their unique role in enhancing the emotional state and self-efficacy. Moreover, both negative affect and self-efficacy mediate the relation between social support and high-calorie food choice, which is consistent with previous studies [14,17]. Thus, good and stable social relationships may contribute to reducing unhealthy eating habits through the two paths of emotion and self-efficacy, which in turn may reduce the risk of developing an eating disorder.

Additionally, although there is evidence that social support contributes to increased healthy eating and decreased rates of unhealthy food intake [15,35,36,37],, we did not find an interaction between group and food type in either experiment. We speculate that this may be because individuals’ perception of reward signals for food are compromised under stress, and that social support provides a buffer that allows individuals to reduce their total craving for rewards. Furthermore, we found the main effect of food type was that participants chose more low-calorie foods than high-calorie foods. These results may have been driven by the fact that the high-calorie foods are visually more satiating and therefore make individuals chose fewer portions in terms of serving size.

Furthermore, this study explored the effect of social support on reward sensitivity using the FID task. However, we did not find any significant result. Possible reasons for these findings include: first, the incentive delay task was originally designed primarily for application in reward anticipation studies using functional magnetic resonance [38]. The incentive delay task belongs to the instrumental conditioning task paradigm, which was proposed based on the animal neurophysiological model [39]. Incentive delay tasks can be insensitive to response time variables. Most studies in the field of eating behavior have not found significant effects on reaction time variables [40,41,42]. For example, the earliest study did not find any difference in reaction time between obese and control groups [43]. In addition, there was no significant effect on reaction time for the incentive delay task using a tasty snack stimulus [30,44]. However, an experimental study has showed that both socially isolated and fasting individuals activated the same brain regions when craving foods or social connections, predicting that these two rewards might share cognitive processing mechanisms [45]. This may suggest an overlap in the processing loops of food reward and social reward, providing a basis for alternative hypotheses of neural mechanisms. Therefore, we hypothesized that functional MRI experiments will help us to answer this question, which may reveal differences in brain activity, and this is what we are doing to further our work.

These findings have important theoretical contributions. First, across our studies, we demonstrated that receiving support from a best friend can change our cognition and behaviors towards healthy eating. Considering the previous mixed results on the effect of social support manipulation [15], this is the first article, to our knowledge, to provide experimental evidence confirming the mechanism of the psychological impact of social support on stress-induced overeating behavior. By doing so, we offer a more profound perspective on the ways in which social support influences eating behavior when coping with stress. Second, we distinguished the effects of social distance, showing the unique role of support from best friends as distinct from support provided by unfamiliar individuals, such as staff. By doing so, we demonstrated the important effects of close social relationships. Compared to strangers, support from a best friend is more effective in increasing emotional state, self-efficacy, and reducing the volume of food choice. Third, we showed that emotion and self-efficacy are the key drivers of our findings, with inhibitory effects on high-calorie food choice. Our findings contribute to work showing the positive effects of emotional state and self-efficacy on health-related outcomes [14,17], and add to the rich understanding of the mechanisms in which social support can hinder unhealthy eating behaviors under acute stress.

### Limitations and Implications

Our study had several limitations. First, the participants were young college students. Although the college-age sample is particularly relevant because of the increased prevalence of eating disorders among college students [46], our results need to be validated in other age groups or clinical samples. Second, cross-sectional and longitudinal studies have shown that social support is a key protective factor for unhealthy eating behaviors in women rather than in men [9,47]; however, we did not consider sex differences owing to the limited sample size. Third, the level of thirst has not been controlled, which may have affected the selection and consumption of low-calorie foods (most of them are juicy).

Our study has implications for resisting stress and binge eating in the current pandemic environment. Receiving social support from friends may alleviate emotional and eating problems, which in turn may prevent problematic conditions, such as obesity and eating disorders. In the context of the current pandemic, encouraging individuals to adopt alternative means of communication to increase access to social support would contribute to the physical health of individuals.

## 5. Conclusions

Overall, the present study elucidated the mechanisms by which social support influences stress-induced overeating behaviors. It also suggests a referential protective factor for people’s physical and mental health in the current unpredictable social environment, as well as providing ideas for therapeutic interventions for clinical eating disorders.

## Figures and Tables

**Table 1:** The description and differences of demographic variables and psychometric measures in Experiment 1.

Characteristics	Friend			Stranger			Decrease			Look			Statistics	
	Range	Mean	SD	Range	Mean	SD	Range	Mean	SD	Range	Mean	SD	*F*	*p*
Age	18.01–25.22	20.34	1.33	18.44–23.86	21.47	1.48	0.93–23.94	19.91	3.72	18.32–27.9	21.06	1.73	3.33	0.02
Hunger (z-score)	0–2.92	−0.11	1	0–2.97	0.07	0.99	0–2.97	−0.09	1.02	0–2.92	0.18	1.02	0.66	0.57
BMI	15.58–28.93	21.09	3.41	15.62–31.56	21.43	3.24	14.7–32.92	20.94	3.73	16.33–28.04	21.03	2.45	0.15	0.92
MSPSS	2–4	2.94	0.64	1.58–3.41	2.63	0.49	0.41–3.58	2.58	0.77	2–4	2.73	0.66	2.01	0.11
PSS	2.07–3.71	2.77	0.41	1.71–4.14	2.79	0.50	1.92–4.35	2.81	0.58	1.92–3.85	2.80	0.48	0.03	0.98
NA (trait)	1.72–3.81	2.58	0.57	1.36–3.81	2.16	0.61	1.45–4.18	2.31	0.63	1.27–3.81	2.37	0.60	2.81	0.04
PA (trait)	1.66–4.66	3.36	0.59	1.77–4.44	3.19	0.65	1.88–4.55	3.15	0.65	2–4.88	3.40	0.59	1.29	0.27
NA (T0)	1.09–3.81	2.11	0.62	1–3.63	1.77	0.63	1–3	1.72	0.48	1–4.09	2.02	0.73	3.13	0.02
PA (T0)	1.66–4	3.20	0.51	1.55–4.44	3	0.75	1.77–4	2.98	0.52	1.55–4	2.95	0.66	1.11	0.34
Self-efficacy	2.4–4.6	3.48	0.56	2.4–4.1	3.42	0.51	1.4–4.7	3.29	0.72	2.3–4.4	3.44	0.43	0.70	0.55
EE	1.33–3.66	2.17	0.58	1–4	2.54	0.65	1–4	2.37	0.81	1–4	2.39	0.74	1.67	0.17
UE	1.22–3	2.28	0.44	1.33–3	2.42	0.37	1.66–3.44	2.31	0.44	1.22–3.55	2.42	0.62	0.81	0.49
CR	1.28–3.42	2.19	0.45	1.16–3.83	2.58	0.63	1–3.83	2.45	0.74	1.28–3	2.35	0.53	2.58	0.06

Note: BMI, Body Mass Index; MPSS, Multidimensional Scale of Perceived Social Support; PSS, perceived stress scale; NA, negative affect; PA, positive affect; T0, baseline; EE, emotional eating; UE, uncontrolled eating; CR, cognitive restraint.

**Table 2:** The description and differences of demographic variables and psychometric measures in Experiment 2.

Characteristics	Friend			Stranger			Decrease			Look			Statistics	
	Range	Mean	SD	Range	Mean	SD	Range	Mean	SD	Range	Mean	SD	*F*	*p*
Age	18.82–23.61	21.20	1.37	18.44–23.86	21.43	1.51	18.59–23.54	20.37	1.35	18.57–24.36	21.22	1.52	3.45	0.02
Hunger (z-score)	−1.58–1.32	−0.26	0.90	−1.18–1.79	0.10	0.99	−1.18–1.79	−0.13	1.03	−1.58–1.32	0.18	1.05	1.42	0.24
BMI	16.73–32.49	21.07	3.47	15.63–31.56	21.48	3.29	15.63–30.12	21.05	3.19	17.01–24.25	20.10	1.80	1.26	0.29
MSPSS	0.83–3.67	2.74	0.56	1.58–3.42	2.63	0.51	0.42–3.58	2.64	0.79	1.17–3.75	2.57	0.65	0.46	0.71
PSS	1.86–3.79	2.79	0.52	1.71–4.14	2.80	0.52	1.93–4.36	2.74	0.59	1.93–3.57	2.69	0.44	0.33	0.80
NA (trait)	1.18–3.91	2.45	0.72	1.36–3.82	2.17	0.63	1.45–4.18	2.24	0.62	1.27–3.91	2.44	0.66	1.57	0.20
PA (trait)	1.67–4.22	3.12	0.60	1.78–4.44	3.18	0.67	1.89–4.56	3.22	0.66	2–5	3.30	0.65	0.49	0.69
NA (T0)	1–3.73	1.83	0.79	1–3.64	1.78	0.65	1–3	1.66	0.50	1–3.17	1.78	0.57	0.40	0.75
PA (T0)	1.44–4.44	2.89	0.80	1.56–4.44	3.02	0.77	1.78–4	3.01	0.55	1.33–4.33	3.20	0.68	1.12	0.34
Self-efficacy	2.1–4.4	3.39	0.55	2.4–4.1	3.44	0.51	1.4–4.7	3.40	0.71	2.3–4.1	3.43	0.48	0.05	0.99
SR	0.29–0.96	0.59	0.17	0.21–0.83	0.55	0.14	0.21–0.83	0.53	0.14	0.21–0.88	0.53	0.17	1.13	0.34
EE	1.33–4	2.41	0.66	1–4	2.52	0.66	1–4	2.43	0.79	1–4	2.17	0.81	1.40	0.25
UE	1.56–3.56	2.31	0.54	1.33–3	2.42	0.38	1.67–3.44	2.36	0.43	1.11–3.44	2.03	0.54	4.48	0.00
CR	1–3.29	2.10	0.59	1.17–3.83	2.59	0.65	1–3.83	2.52	0.69	0.86–2.86	1.81	0.47	12.49	0.00

Note: BMI, Body Mass Index; MPSS, Multidimensional Scale of Perceived Social Support; PSS, perceived stress scale; NA, negative affect; PA, positive affect; T0, baseline; SR, sensitivity of reward; EE, emotional eating; UE, uncontrolled eating; CR, cognitive restraint.

## Data Availability

The datasets analyzed for the current manuscripts are available from the corresponding author upon reasonable request. The lead author has full access to the data reported in the manuscript.

## Supplementary Materials

The following supporting information can be downloaded at: https://www.mdpi.com/article/10.3390/nu15183898/s1, Method S1: Measurements; Method S2: Materials for Social Support Manipulation [48,49,50,51,52,53,54].
