# Supplementary material for: Support from a Best Friend Makes People Eat Less under Stress: Evidence from Two Experiments
Source: Nutrients. 2023 Sep 7;15(18):3898. doi: 10.3390/nu15183898 (PMC10537042; doi:10.3390/nu15183898)
Supplement: Supplementary file 1 [file nutrients-15-03898-s001.zip › nutrients-2567352-supplementary.pdf]

Support from Best Friend Makes People Eat Less under Stress: Evidence from Two Experiments

Supplementary Material

Method S1

Measurements

Body mass index (BMI). Participants self-reported their body height (in m) and weight (in kg). Based on this self-reported information the body mass index (kg/m^2) was calculated.

Eating behaviors. The Three-Factor Eating Questionnaire (TFEQ-R18) is a scale that measures three domains of eating behavior: cognitive restraint (e.g., "I deliberately take small helpings to control my weight"), uncontrolled eating (e.g., "Sometimes when I start eating, I just can't seem to stop") and emotional eating (e.g., "I start to eat when I feel anxious") [48]. The items are measured using a 4-point response scale (1 = definitely false, 4 = definitely true). Cronbach's coefficient α of the TFEQ in this study ranged from 0.78 to 0.85.

Trait perceived social support. The Multidimensional Scale of Perceived Social Support (MSPSS) [49] used 12 item to assesses participants' perceived social support (e.g., "when I have problems, some people [i.e., relatives, friends, classmates] were there accompanying me"). Responses were rated using a 5-point Likert-type scale (1 = never to 5 = always). Cronbach's coefficient α of the MSPSS in the current study was 0.86.

Trait/state perceived stress. Trait perceived stress level was filled in during the first experiment and measured using the Perceived Stress Questionnaire (PSQ) [50], which contains a total of 14 questions (e.g., how often did you feel upset about something unexpected happening in the last month?), rated on a 5-point scale from 1 (never) to 5 (always). Cronbach's coefficient α of the PSQ in the current study was 0.87. State perceived stress was measured by three items (feeling of anxiety/worried/fearful) [4], and it's Cronbach's coefficient α in this study ranged from 0.81 to 0.89.

Trait/state positive and negative emotions. The Positive and Negative Affect Schedule (PANAS) [51] was used to measure trait and state levels of 9 positive emotions (e.g., energetic) and 11 negative emotions (e.g., distracted) with ratings ranging from 1 (almost none) to 5 (extremely many), where alertness was classified from positive emotions in the original version to negative emotions in this study because of the negative meaning in the Chinese culture. Trait positive-negative mood was measured in Session 1, where participants responded based on their emotional state in the most recent month. And the state mood level was measured in Session 2, where participants responded based on their current mood state. Cronbach's coefficient α of the PANAS in this study ranged from 0.87 to 0.90.

Trait Self-efficacy. Self-efficacy was measured using the General Self-Efficacy Scale (GSES) [52]. It contains 10 questions about the individual's self-efficacy when encountering setbacks or difficulties. For example, "I can always find a solution to a problem when I encounter it". A scale of 1 (not at all correct) to 4 (completely correct) on a 4-point Likert scale was used. Cronbach's coefficient α of the GSES in the current study was 0.87.

Reward sensitivity. Reward sensitivity was measured using the Sensitivity to Punishment and Sensitivity to Reward Questionnaire [53]. The reward sensitivity subscale contains 24 questions (e.g., Does encouragement from friends or family enable you to perform well at work and school?), including a 0 (no) and 1 (yes) secondary rating. Cronbach's coefficient α of the questionnaire in the current study was 0.73.

Method S2

Materials for Social Support Manipulation

Stimulus sentences for social support. Twenty-four different Chinese statements adapted from the previous study [54] were used to instruct reappraisal for the interpersonal groups (*Friend* & *Stranger*). These reappraisal statements were rated online by an independent sample (N= 39; 23 females; M_{age}= 25.96 years, SD = 3.17). Participants were asked to imagine themselves under the pressure of preparing for a presentation task and respond to valence ("How do you feel about this statement?"), arousal ("How arousing is this statement?"), social proximity ("How close would you feel to someone who would say this to you?"), effectiveness in relieving stress ("To what extent did this statement relieve you of stress?"), and effectiveness in enhancing self-efficacy ("To what extent does this statement increase your confidence?") on a Likert scale from 1 to 100 (very negative/very calm/very distant/very ineffective/very discouraging to very positive/very exciting/very close/very effective/very encouraging). A total of 28 support statements were rated on validity (M = 57.74, SD = 4.57), arousal (M = 54.12, SD = 5.32), social proximity (M = 59.82, SD = 4.22), effectiveness in relieving stress (M = 58.37, SD = 4.86), and effectiveness in enhancing self-efficacy (M = 54.97, SD = 5.69) dimensions were scored. We excluded four sentences that scored below 1.5 standard deviations from the mean on either dimension, resulting in 12 emotional support sentences (e.g., "Everything will be fine.") and 12 cognitive support sentences (e.g., "Deep breathing will help you.").

Pictures for social support. Digital photos of the best friends were taken (in a dark blue shirt against a white wall, covering the face to mid-chest, without jewelry), and used in the *Friend* group. In addition, a picture of a female or male person (unknown to the participant) was used in the *Stranger* group. The sex of the stranger was matched to the participant to reduce possible gender effects. Scrambled versions of these pictures were created and presented during the non-social groups (*Look*, which was the control group, and the intrapersonal group, here simply referred to as *Decrease*) to match the trial structure of the interpersonal groups.
